# Antioxidative properties of *Ocimum gratissimum* alters Lead acetate induced oxidative damage in lymphoid tissues and hematological parameters of adult Wistar rats

**DOI:** 10.1016/j.toxrep.2021.01.003

**Published:** 2021-01-10

**Authors:** John Chukwuma Oyem, Lilian Ebite Chris-Ozoko, Mamerhi Taniyohwo Enaohwo, Francisca Osamahemwem Otabor, Vera Anieze Okudayo, Onoriode Andrew Udi

**Affiliations:** a Department of Human Anatomy and Cell Biology, Faculty of Basic Medical Sciences, Delta State University, Abraka, Nigeria; b Department of Basic Medical Sciences (Anatomy Unit), College of Natural and Applied Sciences, Achievers University Owo, Ondo State, Nigeria

**Keywords:** BW, Bodyweight, CAT, Catalase, GSH, Glutathione, Hb, Hemaglobin, H_2_O_2_, Hydrogen peroxide, LA, Lead Acetate, MDA, malondialdehyde, OG, *Ocimum gratissimum*, ROS, Reactive Oxygen Species, RBC, Red Blood Cell, SOD, Superoxide Dismustase, WBC, White Blood Cell, Oxidative stress, Antioxidation, *Ocimum gratissimum*, Lead acetate, Spleen, Thymus

## Abstract

Lead exposure is a well-known environmental hazard. Its accumulation in humans may pose a danger to health. The present study investigated the beneficial effect of *Ocimum gratissimum* extract (OG) in reducing lead acetate (LA) induced oxidative damage in the spleen, thymus, and hematological indices. We employed an *in vivo* model of LA induced Wistar rats and administered 125 mg/kg/bw and 250 mg/kg/bw of OG extracts respectively. Our control groups were divided into 2; the first group received normal saline, feed, and water while the second group was administered OG extracts only. We assessed the levels of malondialdehyde (MDA), glutathione (GSH), superoxide dismutase (SOD), and catalase (CAT) in the thymus and spleen and estimated percentages of blood cells. Our results showed that LA induces oxidative damage by significantly elevating MDA and diminishing GSH levels, SOD, and CAT activities. LA administration led to a significant decline in blood parameters. However, co-administration with OG compensated oxidative stress by significantly reducing MDA, increasing GSH, SOD, and CAT. Oral administration of OG to rats attenuated anemia, thrombocytopenia, leucocytosis, eosinophilia, monocytosis, and neutropenia induced by LA. The present study indicates that LA induced Spleen, thymus, and blood toxicity, which was reversed by oral OG administration.

## Highlights

• Chronic lead toxicity was induced in male rats by oral administration of lead acetate.
• Effect of *Ocimum gratissimum* in lead acetate toxicity on spleen, thymus, and blood.
• Oral lead acetate administration led to oxidative damage in spleen, thymus and blood.
• *Ocimum gratissimum* extract reversed oxidative stress and enhanced antioxidant enzymes.
• *Ocimum gratissimum* averts lead acetate-induced toxicity in the blood, thymus and spleen.

## Graphical abstract

**Figure:**
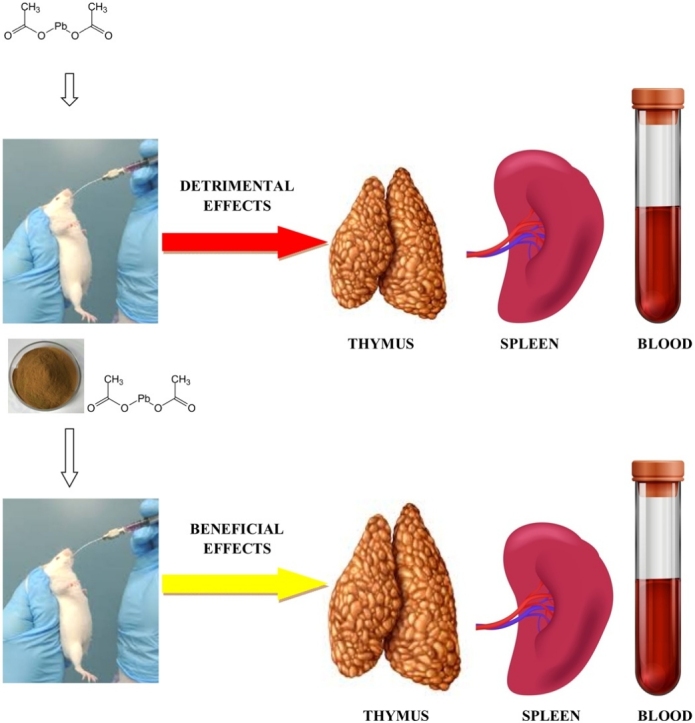

## 1 Introduction

Lead is a non-biodegradable toxic element that can cause acute and severe cardiovascular, hematological, reproductive, digestive, immunological, and neurodegenerative diseases [[1], [2], [3]]. To date, different industries apply lead in the productions of cars, paint, pottery, and plastic materials [1] while in modern medicine, it is used as an astringent.

Lead constitutes one of the major environmental pollutants in developing countries [[4], [5], [6]]. According to Alwaleedi [7], lead enters the human body through inhalation, skin, and the alimentary tract. Lead levels in the blood of about 40–60 ug/dL are considered very toxic [8]. A suspected lead poisoning case was reported in Nigeria in Unguwan communities, Niger state, Nigeria [9]. Among the 48 affected individuals were mostly children with blood lead levels of 171.5–224 mg/dL including 14 deaths were reported [9]. Another study conducted in Jos, Nigeria, documented blood lead levels were higher in Muslim subjects, those whose homes are situated close to places where car cells were melted and those using eye cosmetics [10].

Although the mechanism of action of lead is not well elucidated, the prime targets of lead toxicity including heme synthesizing enzymes, thiol-containing antioxidants and oxidative enzymes (superoxide dismutase (SOD), catalase (CAT), glutathione peroxidase, glucose 6-phosphate dehydrogenase and glutathione (GSH)) [11]. Low concentrations of lead in the blood, inhibits the action of oxidative enzymes thus leading to oxidative stress [1,2,11,12]. A prime biomarker of lead oxidation is lipid peroxidation [1,5,13]. The generated free reactive oxygen species traps electrons from intracellular lipids thus leading to cellular damage [1]. Lead induces oxidation of hemoglobin (Hb), which leads to Red Blood Cells hemolysis (RBC) [1].

Lead directly affects the hematopoietic system by altering the production of hemoglobin through the inhibition of various key enzymes involved in the heme synthesis such as cystolic ***δ-aminolevulinic acid dehydratase (ALAD)***, which catalyzes porphobilinogen formation from *δ*-aminolevulinic acid (ALA), ***aminolevulinic acid synthetase (ALAS)***, a mitochondrial enzyme that catalyzes aminolevulinic acid (ALA) formation, and, ***ferrochelatase*** a mitochondrial enzyme that catalyzes iron insertion into protoporphyrin during heme formation [14,15].

Globally, the antioxidative properties of various plants have been applied in the management of diseases especially in developing countries where they have been documented in traditional medicine [13,[16], [17], [18], [19], [20],^21^]. About 80 % of individuals from developing countries are using traditional medicines to meet up their primary health care needs [21]. *Ocimum gratissimum* (OG) commonly called scent leaf is a dietary culinary spice used by different ethnic groups in Nigeria and other countries. It is called “Arurunta” by the Ukwuanis, “Ebe-amwonkho” in Edo, “Tchayo” in Fon, “Efinrin” in Yoruba, “Daidoya” in Hausa, “Nchuanwu” in Igbo, “Ntonng” in Ibibio, “Kunudiri” in Okrika and “Nunum’ in Akanb [22,23].

The phytochemical screening of OG revealed that it contains alkaloids, tannins, flavonoids, phytates, and oligosaccharides [[24], [25], [26], [27], [28], [29]]. Matasyoh et al. in 2007, demonstrated the presence of Ocimum oil (essential oil) and non-phlobatannins; Ocimum oil is made up of thymol (48.1 %), as its most constituent, p-cymene (12.5 %) and trace elements. These constituents have been reported to possess several medicinal properties such as antibacterial activities [25,28,30,31] antidiabetic properties [27] and anti-hyperlipidemic effect [22,24]. Also, it was reported to improve blood parameters in experimental animals [23,26,32].

Although researchers have explored the deleterious effect of lead on the human physiology, prevention of lead exposure on humans is yet to be achieved [1,33]. Suradkar et al. [34], observed degeneration and necrosis of the splenic cells following a 28-day administration of lead acetate while Ekanem et al. [35], reported splenomegaly, a significant reduction in packed cell volume (PCV) and minimal change in the hemoglobin levels in Wistar rats administered lead acetate. A special paper by [36], reported a reduction in RBC count, and Hb White Blood Cell (WBC) count. Similar experimental studies revealed that lead acetate increased malondialdehyde (MDA) and reduced antioxidant enzyme activities in the bone marrow [37,38]. A more recent study by Okechukwu et al. [26], reported that vitamin C and OG restored the distortive histoarchitecture of thymus induced by lead.

Several studies have x-rayed critically the deleterious effects of lead acetate on human physiology [[1], [2], [3]]. Scant literatures have evaluated the effect of lead acetate on the blood, spleen and thymus histology as well as the roles of OG in ameliorating the deleterious effect of LA-induced toxicity [26,34,35]. According to this background, we designed the current study to determine the therapeutic effect of OG against Lead acetate (LA) induced oxidative damage on the thymus, spleen, and hematological parameters.

## 2 Materials and methods

### 2.1 Animal care

The Research Ethics Committee on Animal Use of Delta State University Abraka, Nigeria approved every protocol and procedure used with reference number (DELSU/CHS/ANA/12/40), in line with Animal Reforms Guidelines and the National Institute of Health Guide for Care and Use of Laboratory Animals.

### 2.2 Drugs

LA of 100 % purity manufactured by BDH chemical ltd. England was purchased from a chemical shop in Onitsha, Anambra State, Nigeria. The dosage of LA (120 mg/kg/bw) administered was adopted from Suradkar et al. [34].

## 3 Plant material

Fresh OG leaves were obtained from a farmland in Owo, Ondo State, Nigeria. The leaves were identified and authenticated by a plant curator in University Herbarium, Ado-Ekiti, Nigeria with herbarium number (UHAE2019155).

### 3.1 Plant extracts preparation

Fresh leaves of OG were air-dried in the laboratory at ambient temperature (30 ± 2°c) for three weeks, and was pulverized using a laboratory mechanical grinder. 247 g of the powdered sample was extracted using 4400 mL of 50 % ethanol (*via* cold maceration) for 48 h. The mixture were decanted and filtered using sterile Whatman paper (3 mm) to obtain a semi-solid residue which was further placed in a sterile glass dish in a desiccator for complete dryness. The extract was reconstituted in water and was administered orally. The dose of OG administered was based on result obtained from our preliminary investigation and that from Mohammed et al. [39], who reported an LD_50_ of LA as 1264.9 mg/kg.

### 3.2 Experimental design

Thirty (30) adult male Wistar rats (∼200 g; Delta State University, Abraka Animal Holdings) were housed under laboratory conditions of humidity, temperature, and unrestricted access to rat chow pelletized meal and water. The animals were left to acclimate. Following a two days acclimation, experimental animals were randomly assigned into 5 groups (control, OG, LA, LA + OG_1_, LA + OG_2_) and placed in animal house maintained under controlled conditions of temperature (23 ± 2 °C) and humidity (50 ± 5 %) and a 12-h light-dark cycle. Animals in the control group were administered normal saline and had *ad libitum* access to drinking water and food only unlike other experimental animals who received food and water freely together with the test compounds. The OG group received 250 mg/kg/bw of aqueous leaf extract of OG only throughout the experiment, the LA group received 120 mg/kg/bw of LA throughout the experiment while group LA + OG_1_ and group LA + OG_2_ received 120 mg/kg/bw of LA +125 mg/kg/bw of OG and 120 mg/kg/bw of LA +250 mg/kg/bw of OG, respectively for 28 days (each for 14 days respectively) see Table 1 below. All test compounds were administered orally once a day (8:00 am) with the use of an orogastric tube. Specific concentration of OG administration was chosen based on the fact that the LD50 of OG is calculated to be 1264.9 mg/kg body weight [40].

**Table 1:** Experimental design for pre-treatment with ethanolic extract of OG before oral lead acetate administration.

Groups	Dosage
Control (n = 6)	Animals were administered 0.1 mL orally of 0.9 % normal saline orally for 28 days.
OG extract group only (n = 6)	Animals were administered 250 mg/kg/bw of OG extract only orally for 28 days.
LA group only (n = 6)	Animals were administered 120 mg/kg/bw of LA only orally for 28 days.
LA + OG1 (n = 6)	Animals were administered 120 mg/kg/bw of LA orally for 14 days followed by 125 mg/kg/bw of OG extract orally for 14 days
LA + OG2 (n = 6)	Animals were administered 120 mg/kg/bw of LA orally for 14 days followed by 250 mg/kg/bw of OG extract orally for 14 days

Key; OG: *Ocimum gratissimum*; LA: Lead acetate; bw: body weight.

The Percentage extraction yield for *Ocimum gratissimum* extract was calculated using [mass of extract (g)/mass of plant sample (g)] × 100 stated by Okoduwa et al. [27]. Mass of extract was 20 g and mass of plant sample was 500 g, which yielded 4 g/100 g.

### 3.3 Blood collection

After 28 days of administration, blood was collected through the retro-orbital vein into a dipotassium EDTA bottle. It was well mixed with the anticoagulant to prevent coagulation.

### 3.4 Animal sacrifice

Animals were sacrificed by cervical dislocation. The spleen and thymus tissues were harvested, homogenized, and used for biochemical analysis.

### 3.5 Biochemical assessment

Biochemical parameters were assayed using homogenates from the spleen and thymus.

#### 3.5.1 Malondialdehyde (MDA) level assessment

This was used to determine lipid peroxidation and was determined quantitatively by measuring MDA content using the Tsika’s method [41]. In this test, the reaction mixture contained 1.0 mL tissue homogenate, 1.0 mL of TCA (10 %), and 1.0 mL TBAR (Thiobarbituric acid) (0.67 %). Test tubes were placed in a boiling water bath for 45 min, and were shifted to ice bath. The tubes were then centrifuged at 2500×g for 10 min. The malondialdehyde (MDA) levels formed in each of the samples were calculated by measuring the optical density of the supernatant at 532 nm. The results were expressed as the nmol MDA formed/gram tissue by using a molar extinction coefficient of 1.56 × 105 M^−1^ cm^−1^ [41] (Table 2, Table 3).

**Table 2:** The effect of *Ocimum gratissimum* on lead induced toxicity of Wistar rats’ hematological parameters.

Groups	PCV (%)	Hb (g/L)	WBC(x10^9^/L)	RBC (10^12^/L)	Platelet(10^9^/L)
CTRL	44.7±2.73	14.0±0.15	5.3±0.22	7.4±0.33	301±2.40
OG	48.3±1.21^a^	15.1±0.12^a^	4.1±0.12^a^	7.7±0.15^a^	170±9.13^a^
LA	27.0±1.47^a^	9.0±0.15^a^	4.4±0.15^a^	4.5±0.09^a^	146±0.91^a^
LA + OG_1_	46.0±1.00^b^	14.5±0.80^b^	7.0±0.58^b^	7.5±0.10^b^	190±1.53^b^
LA + OG_2_	45.0±1.20^b^	14.2±0.10^b^	5.30±0.70^b^	7.60±0.23^b^	209±2.30^b^

Data were expressed as mean ± SD.

^ab^Significant different when compared to the control, OG and LA group (p < 0.05) (n = 5).

a Significant difference when compared to the control group (p < 0.05).

b Significant difference when compared to the lead acetate treated group.

**Table 3:** The effect of *Ocimum gratissimum* on lead-induced toxicity of Wistar rats’ white blood cell differential counts.

Groups	Eosinophils(%)	Monocytes (%)	Lymphocyte (%)	Neutrophils(%)
CTRL	5.70±0.82	5.60±1.00	29.0±1.00	70.3±0.90
OG	3.60±0.90^a^	6.70±0.38^b^	18.7±0.90	71.0±2.30^a^
LA	6.30±0.90^a^	5.30±0.90^a^	23.3±0.90	68.7±1.86^a^
LA + OG_1_	5.70±1.20^ab^	4.00±0.58^ab^	23.3±0.70	70.0±2.3^ab^
LA + OG_2_	5.70±0.90^ab^	4.70±0.33^ab^	24.3±0.90	71.0±2.6^ab^

Data were expressed as mean ± SD.

a Significant difference when compared to the control group (p < 0.05).

b Significant difference when compared to the lead acetate treated group.

ab Significant difference when compared to the control, OG extract and LA group (p < 0.05) (n = 5).

#### 3.5.2 Reduced glutathione (GSH) levels assessment

Part of the tissues were washed briefly in saline, and then homogenized in an ice-cooled buffer that is composed of 1.15 % KCl, 0.01 M sodium phosphate buffer pH 7.4. Concentrations of GSH were determined using method described by Ellman [42].

#### 3.5.3 Assessment of catalase (CAT) activity

This was determined using the method adopted by Chia with slight modifications [43]. 600 μL of 0.1 M PBS (pH 7.1) was added to 350 μL of 0.059 M hydrogen peroxide (H_2_O_2_) and 1.0 mL of homogenate. The absorbance was read by measuring the optical density of the supernatant at 340 nm.

#### 3.5.4 Measurement of superoxide dismutase (SOD) activity

SOD was evaluated following the method described by Sirota [44]. This method was based on the ability of SOD to inhibit the autoxidation of epinephrine at pH 10.2.

### 3.6 Assessment of hematological parameters

The RBC count, Pack Cell Volume (PCV)), WBC count, monocytes, lymphocytes, neutrophils, eosinophils were adopted from the Hemocytometer method of Thrall et al. [45]. Hemoglobin (Hb) concentration was measured using the Cyanmethemoglobin method of Higgins [46].

#### 3.6.1 Statistical analysis

**Fig. 1:**
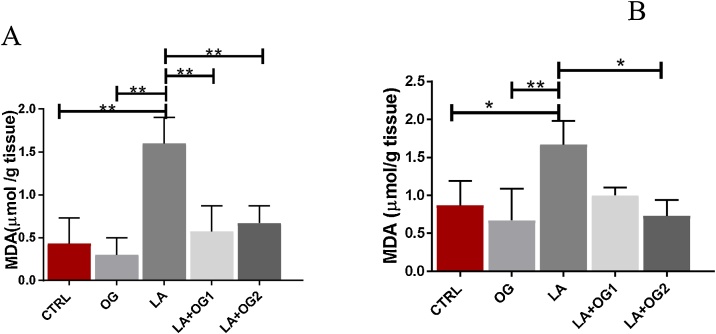
The effect of *Ocimum gratissimum* extract on MDA levels of LA induced toxicity on the spleen and Thymus.

Data obtained from hematological and biochemical analyses were subjected to Statistical Package for Social Sciences, version 23 (SPSS produced by SPSS Inc. Chicago), and analyzed using one-way analysis of variance (ANOVA). Tukey’s post hoc test was used to determine significant differences within each group. The results were expressed as mean ± standard deviation (mean ± SD) and mean differences were considered significant at *p*< *0.001, 0.01, 0.05* (Fig. 1).

## 4 Results

**Fig. 2:**
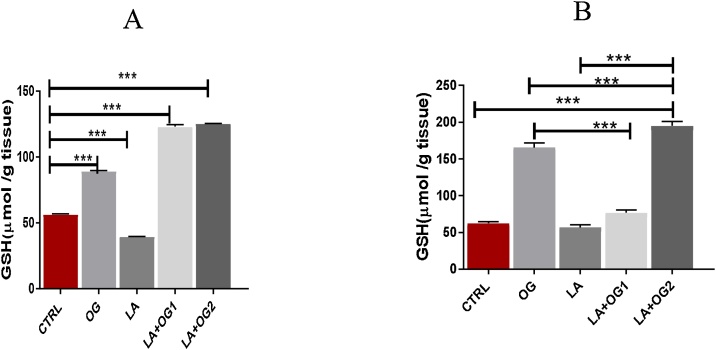
The effect of *Ocimum gratissimum* on Spleen and Thymus glutathione levels.

One-Way ANOVA test of (1a: MDA activities in the spleen, 1b: MDA activities in the thymus) shows a significant increase in spleen and thymus MDA actvities of the experimental rats administered only LA when compared with the control (*p < 0.05, ** p < *0.01);* however, there is a significant decrease in spleen and thymus MDA activities following the administration of varying doses of OG (*p < 0.05, ** p < *0.01*. CTRL: Control group, OG: *Ocimum gratissimum* group, LA: Lead acetate group, LA + OG_1_: Lead acetate + *Ocimum gratissimum* extract (low dose), LA + OG_2_: Lead acetate + *Ocimum gratissimum* extract (High dose) (Fig. 2).

**Fig. 3:**
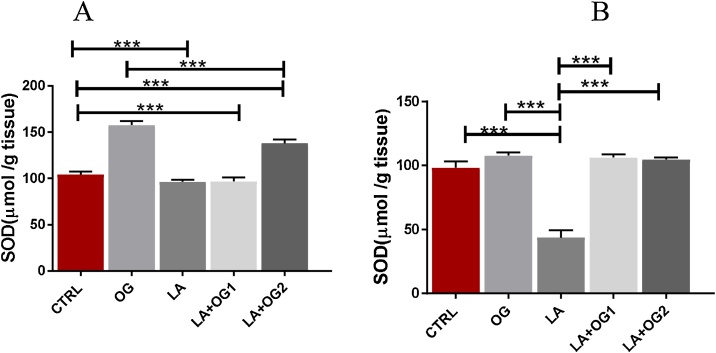
The effect of LA and *Ocimum gratissimum* administration of SOD activities in the spleen and thymus.

A) One-Way ANOVA test shows a significant increase in GSH levels in the spleen of the experimental rats when compared with the control, OG, and LA experimental animals,(*** p < *0.001)*. B) A significant GSH levels were recorded in OG and LA + OG_1_ in the thymus. LA led to a significant increase in GSH levels in the thymus which was further increased following the administration of OG. CTRL: Control group, OG: *Ocimum gratissimum* group, LA: Lead acetate group, LA + OG_1_: Lead acetate + *Ocimum gratissimum* extract (low dose), LA + OG_2_: Lead acetate + *Ocimum gratissimum* extract (High dose) (Fig. 3).

**Fig. 4:**
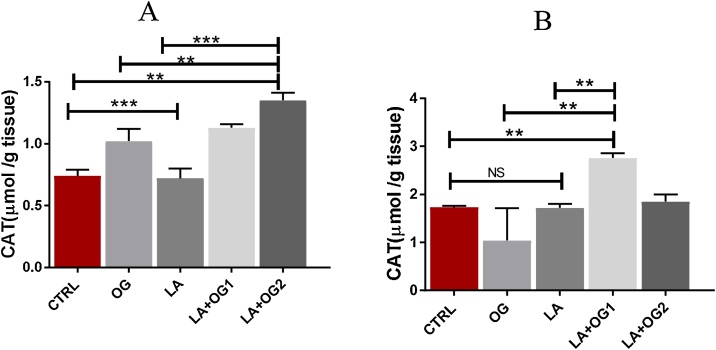
The effect of *Ocimum gratissimum* on CAT activities of LA induced toxicity on the spleen and Thymus.

3a). One-Way ANOVA test shows a significant decrease in the spleen’s SOD activities of the experimental rats when compared with LA mice and a significant increase in SOD levels in the spleen when compared with the OG and CTRL groups, (*** p < *0.001).* 3b). It showed a significant decrease (*** p < *0.001)* in SOD activities in the thymus of experimental animals administered LA only. Further, this SOD activity in the thymus was significantly increased following the administration of OG in groups LA + OG_1_ and LA + OG_2_ animals (*** p < *0.001)*. CTRL: Control group, OG: *Ocimum gratissimum* group, LA: Lead acetate group, LA + OG_1_: Lead acetate + *Ocimum gratissimum* (low dose), LA + OG_2_: Lead acetate + *Ocimum gratissimum* (High dose) (Fig. 4).

One-Way ANOVA test of (4a: CAT activities in the spleen) shows a significant decrease in the spleen’s CAT activities of the experimental rats administered LA + OG_1_ with a significant increase in the CAT activities of animals administered LA + OG_2_ when compared with the control, OG, and LA groups (*p < 0.05, ** p < 0.01***p < 0.001). However, in the thymus (2b) there is a significant increase in CAT activities in the thymus of experimental animals that received LA + OG_1_ when compared with control, OG, and LA groups(*p < 0.05, ** p < 0.01***p < 0.001. However, there is no significant difference in the thymus CAT levels in animals in group LA + OG_2_. CTRL: Control group, OG: *Ocimum gratissimum* group, LA: Lead acetate group, LA + OG_1_: Lead acetate + *Ocimum gratissimum* (low dose), LA + OG_2_: Lead acetate + *Ocimum gratissimum* (High dose).

One-Way ANOVA test shows a dose-dependent significant increase in PCV, WBC, RBC, Hb, and platelet counts of the LA + OG_1_ and LA + OG_2_ animals when compared with the control and experimental group. CTRL: Control group, OG: *Ocimum gratissimum* group, LA: Lead acetate group, LA + OG_1_: Lead acetate + *Ocimum gratissimum* (low dose), LA + OG_2_: Lead acetate + *Ocimum gratissimum* (High dose).

Treatment of experimental animals with lead acetate caused a significant (p < 0.05) decrease in neutrophil and monocytes and a significant increase in eosinophils when compared with the control. These decrease in neutrophil and monocyte were significantly increased and normalized (p < 0.05) in animals that received LA + OG_1_ and LA + OG_2_ treatment respectively. There was no recorded significant effect on lymphocyte percentage. CTRL: Control group, OG: *Ocimum gratissimum* group, LA: Lead acetate group, LA + OG_1_: Lead acetate + *Ocimum gratissimum* (low dose), LA + OG_2_: Lead acetate + *Ocimum gratissimum* (High dose).

## 5 Discussion

Even though the ubiquitous nature of lead is beneficial to man, it is still considered an environmental toxin [1]. There is no safe level of lead exposure in the body physiology. Oxidative stress is one of the prime mechanisms of lead-induced toxicity [1,47]. An experimental study presented evidence that stress conditions disrupt the immune function of humans and animals by modifying the pro-oxidant capacity of neutrophils and macrophages [11]. These cause a decline in the immune response to infections [3,11,37]. Findings from the current study demonstrated that oral administration of OG extract attenuated LA induced oxidative damage on the spleen, thymus and hematological parameters by lowering MDA levels, increasing GSH levels, catalase and SOD activities and by improving blood parameters.

Lead-induced oxidative damage involves firstly, the Reactive Oxygen Species (ROS) generation such as singlet oxygen, hydrogen peroxide (H_2_O_2_), and hydroperoxide, and secondly, the destruction of the body’s antioxidant defense system [48]. Due to the reported cases of rapidly increasing levels of lead poisoning in Nigeria, our study was designed to determine the controversial role of lead-induced toxicity concerning its relationship to oxidative damage in spleen, thymus and hematological parameters as well as assessing the roles of OG in interfering with oxidative damage induced by lead.

We hypothesized that OG extract may alter lipid peroxidation by reducing the levels of MDA. In our study, we exposed rats to OG extract, LA, LA + doses of OG extract, and assessed the levels of MDA in the spleen and thymus. Our study revealed that LA caused a significant increase in the MDA levels, which was decreased following co-administration with low and high doses of OG extract. This implied that OG extract attenuates oxidative damage induced by LA in the thymus and spleen by lowering MDA. One of the reasons for increasing levels of MDA in the spleen is that high levels of ROS transverses the cell membrane and destroys neighboring cells which result in an increase in the ROS that facilitates the cellular damage of the spleen [2]. Findings from this study are in line with that of Okechukwu et al. [26], who demonstrated that aqueous extract OG possesses a similar mechanism with vitamin C, in restoring the histoarchitectural details of the thymus of animals exposed to LA.

For the SOD antioxidant system assay, our findings revealed that oral administration of OG extract at low and high doses compensated the activities of SOD in experimental animals administered LA and OG extract. This observation indicated that the chemical constituents of OG extract activated SOD isoenzyme activity, which ameliorated oxidative damage induced by LA in the thymus and spleen. It was clearly shown by the dose significant response in the activity of SOD of animals in groups LA + OG_1_ and LA + OG_2_ when compared to LA and control groups. One of the mechanisms for this finding could be that SOD is an inhibitory agent of neutrophil-mediated inflammation and may be a viable therapeutic target for neutrophil induced ROS-dependent tissue damage [49]. Several studies believed that SODs form a very strong antioxidant defense system against oxidative stress and serves as an anti-inflammation agent in the prevention of precancerous progression and hemoglobinopathies [[49], [50], [51], [52], [53], [54]].

Redox biomarkers such as GSH and CAT have been implicated in understanding the mechanisms related to the action of mixtures of xenobiotics on animal oxidative profile based on the current toxicological approach termed "the real-life exposure scenario” [55]. In the present study, we investigated the role of GSH and CAT, a powerful antioxidant for disrupting ROS in lead-induced toxicity in the spleen and thymus. LA administration led to a significant decrease in GSH levels in both organs. However, following oral administration of low and high doses of OG extract to LA induced animals led to a dose dependent significant increase in levels of GSH. This implied that exogenous administration of OG extract increases GSH antioxidant levels, which was depleted by LA. This is really a significant observation since the elevated increase of GSH in OG groups attenuated LA-induced impairment in the intrinsic antioxidant defense mechanisms. Similar finding was reported by Offor et al. [56], who noted that activated charcoal administration led to a significant increase in GSH levels of LA acetate induced rats. According to Hultberg [57], GSH in cells is rendered inactive by lead which leads to GSH synthesis from cysteine *via* the γ-glutamyl cycle, and it becomes ineffective in restoring the supply of GSH. Moreover, lead inactivates the activities of *δ*-aminolevulinic acid dehydratase (ALAD), glutathione reductase (GR), glutathione peroxidase (GPX) and glutathione-S-transferase, which further depresses the of levels glutathione [58].

Catalase, functions by modulating hydrogen peroxide at the cellular level which implies that the catabolism of CATs is protective [59]. In this study, we quantified and compared the activities of catalase in control, OG, LA, and LA + OGs experimental animals. We discovered a dose dependent significant increase in CAT activity in experimental animals co-administered with OG compared with the control and LA groups. This result pattern thus implies that the oral administration of OG extract at low and high doses increased the activities of CAT already compromised by LA induction. Reduction in the activity of CAT causes the generation of Hydrogen peroxide (H_2_O_2_) in cells [59]. Further, H_2_O_2_ associates with hemoglobin-containing iron to stimulate the Fenton and Haber-Weiss reactions as well as elevating other ROS activities [60,61],

One of the major functions of the thymus is to promote the development of T-lymphocytes whose major function is cell-mediated immunity [26]. Upon maturation, this T-lymphocytes exits the thymus and is channeled to the lymph nodes and spleen *via* blood vessels [26]. The spleen is a primary lymphoid organ that destroys red blood cells and recycles heme iron [2]. Lead exposure is known to induce anemia, leukocytosis, monocytopenia, polychromatophilia, glycosuria, increased serum urobilinogen, and hematuria while in chronic cases of lead exposure it leads to neutrophilia, leukocytosis, eosinopenia, and monocytopenia. In the current study, we hypothesized that the antioxidative properties of OG extract will ameliorate LA induced blood aberration by administering OG extract orally to LA induced experimental animals for an additional 14 days. Interestingly, our result revealed that oral administration of OG extract following LA exposure led to a significant increase in PCV, RBC, Hb, WBC, and platelet count. This implied that the antioxidative properties of OG extract are potent in averting LA induced anemia, leucocytosis, and thrombocytopenia.

Furthermore, we investigated the effect of LA following OG extract administration on circulating WBC in the spleen and thymus. The spleen is the largest peripheral lymphatic organ which contains about one-fourth of all the lymphocytes in the body [2]. The results regarding the white blood differential count are somewhat surprising giving that oral administration of OG led to a significant elevation in neutrophil count and a significant decline in the percentages of monocytes, and eosinophils with no observable significant changes in lymphocyte count of the experimental animal. Our results indicated that oral administration of OG extract reversed eosinophilia, monocytosis, and neutropenia induced by LA. These findings may also be mediated by the roles of neutrophils in inflammation [62]. Activated neutrophils attach to the endothelial layers of blood vessels and migrate to the extravascular space where ROS, proteases enzymes, and chemokines, which damage normal tissues and extracellular matrix proteins, are released. In addition, O_2_ activates cells of the endothelium which enhances neutrophil infiltration [63].

Lead-induced toxicity on the circulating blood may be mediated by the activities of lead on key enzymes of heme synthesis [1,47]. Lead has also been demonstrated to reduce the life span of RBC in circulation by increasing the vulnerability of their cellular membranes [1]. The consequence of these mechanisms leads to a decrease in RBC (anemia) [2,9,38]. According to Vij [64], lead-induced anemia is divided into; frank anemia which is caused by a prolonged elevation of blood lead level and hemolytic anemia which has been linked to chronic lead exposure.

Although the exact immunomodulatory mechanisms of co-administration of OG extract on LA induced lymphoid toxicity has not been elucidated, our findings propose that OG extract protects the spleen and thymus from lipid peroxidation by increasing and reducing the levels of GSH and MDA respectively, and increasing the activities of antioxidants such as CAT and SOD. This may be attributed to the antioxidative properties of OG extract which has been demonstrated to possess protective properties [23,26,28,30,[65], [66], [67], [68]]. These plant may exert similar protective mechanism of action with flaxseed isolate incorporated with lemon juice which was reported to exert protective properties on lead induced kidney and liver toxicity [69]. Antioxidants prevent lead-induced toxicity by; inactivating the activities of generated ROS at the gene level, lead ion chelating, and prevention of ROS formation in its maintenance in a redox state thus contributing to its weakness in reducing molecular oxygen [65,70,71]. Also, assessing the hazard index (HI) and hazard quotient (HQ) of Lead will shed more light in understanding the level of risk and toxicity of lead in the experiment animals so as to proffer an analysis for the mechanism of any outcomes with exposure to lead and OG [72,73].

## 6 Conclusion

Lead acetate exposure is deleterious in experimental animals including hematological alterations, lipid peroxidation, and disruption of the body’s antioxidant defense system. However, oral administration of OG protected the blood, spleen, and thymus from lead acetate induced oxidative stress. This indicates that the antioxidative properties of OG extract may be a viable therapeutic target in LA-induced blood, spleen and thymus toxicity in Wistar rats. Future studies should explore the exact mechanisms of actions of OG extract in attenuating lead acetate toxicity by carrying out histological, immunohistochemical, and molecular studies. Furthermore, phytochemical screening should be conducted on this agent to ascertain the major chemical compound of OG, which possesses the antioxidant properties.

## CRediT authorship contribution statement

**John Chukwuma Oyem:** Formal analysis, Writing - original draft, Writing - review & editing. **Lilian Ebite Chris-Ozoko:** Conceptualization, Supervision. **Mamerhi Taniyohwo Enaohwo:** Data curation, Validation. **Francisca Osamahemwem Otabor:** Project administration, Methodology. **Vera Anieze Okudayo:** Project administration, Methodology. **Onoriode Andrew Udi:** Investigation, Resources.

## Declaration of Competing Interest

The authors declare no conflict of interest.
